# Correlation of plasma cell assessment by phenotypic methods and molecular profiles by NGS in patients with plasma cell dyscrasias

**DOI:** 10.1186/s12920-022-01346-1

**Published:** 2022-09-23

**Authors:** Ekaterina Rebmann Chigrinova, Naomi A. Porret, Martin Andres, Gertrud Wiedemann, Yara Banz, Myriam Legros, Matthias Pollak, Elisabeth Oppliger Leibundgut, Thomas Pabst, Ulrike Bacher

**Affiliations:** 1 Department of Hematology; Inselspital, Bern University Hospital, University of Bern, Bern, Switzerland; 2 Institute of Pathology, University of Bern, Bern, Switzerland; 3 Center for Laboratory Medicine (ZLM), Inselspital, University of Bern, Bern, Switzerland; 4 Department of Medical Oncology, Inselspital, University of Bern, Bern, Switzerland

**Keywords:** Multiple myeloma, NGS, Bone marrow aspirate, Flow cytometry, Trephine biopsy, Histopathology

## Abstract

**Background:**

Next-generation sequencing (NGS) detects somatic mutations in a high proportion of plasma cell dyscrasias (PCD), but is currently not integrated into diagnostic routine. We correlated NGS data with degree of bone marrow (BM) involvement by cytomorphology (BMC), histopathology (BMH), and multiparameter flow cytometry (MFC) in 90 PCD patients.

**Methods:**

Of the 90 patients the diagnoses comprised multiple myeloma (n = 77), MGUS (n = 7), AL-amyloidosis (n = 4) or solitary plasmocytoma (n = 2). The NGS panel included eight genes *CCND1*, *DIS3*, *EGR1*, *FAM46C* (*TENT5C*), *FGFR3*, *PRDM1*, *TP53*, *TRAF3*, and seven hotspots in *BRAF*, *IDH1*, *IDH2*, *IRF4*, *KRAS*, *NRAS*.

**Results:**

Mutations were detected in 64/90 (71%) of cases. *KRAS* (29%), *NRAS* (16%) and *DIS3* (16%) were most frequently mutated*.* At least one mutation/sample corresponded to a higher degree of BM involvement with a mean of 11% pathologic PC by MFC (range, 0.002–62%), and ~ 50% (3–100%) as defined by both BMC and BMH.

**Conclusions:**

The probability of detecting a mutation by NGS in the BM was highest in samples with > 10% clonal PC by MFC, or > 20% PC by BMC/ BMH. We propose further evaluation of these thresholds as a practical cut-off for processing of samples by NGS at initial PCD diagnosis.

**Supplementary Information:**

The online version contains supplementary material available at 10.1186/s12920-022-01346-1.

## Introduction

Risk stratification in patients with multiple myeloma (MM) has continuously evolved in the last decades [1–3]. However, the duration of response remains highly variable [4–6]. Heterogeneous mutational profiles may, in part, explain such clinically divergent outcomes [7–15].

There appears to be a complex landscape of genetic progression from early plasma cell precursor alterations towards monoclonal gammopathy of unclear significance (MGUS) to, finally, symptomatic MM [8, 15].

According to previous studies on plasma cell dyscrasias (PCD) genomics, the most frequently occurring mutations involve members of the *RAS/MAPK* cell proliferation pathway (in up to 40% of cases) including *KRAS*, *NRAS* and *BRAF* [6, 16], and the *NF-kB* pathway comprising around 20% of all myeloma cases, with *NFKB1*, *TRAF3*, *CYLD*, *LTB* as the most frequently mutated genes [16, 17]. The DNA repair pathway genes (*TP53*, *ATM*, *ATR*) are affected by mutations in around 15% of cases and the cell cycle regulators *RB1 and CCND1* in 5% [16]. The most frequently mutated tumor suppressor genes are *FAM46C* (*TENT5C*) (up to 10%) *and IRF4* (~ 5%) [7, 8, 16, 18–20].

Several next-generation sequencing (NGS)- based studies suggested correlations between genomic, clinical and laboratory features, which could affect prognosis, disease classification and adjustment of therapies, including novel immunologic treatment options [7, 18, 20]. Moreover, screening for molecular mutations may help to identify additional targets to further improve therapeutic options [18, 21].

Next-generation sequencing (NGS) is an established tool for the detection of both somatic as well as germline mutations in neoplasms, and is widely used during the diagnostic work-up of hemato-oncological malignancies [22]. However, the actual costs and the workload of NGS still must be weighed up against the possible clinical benefit for patients with PCD.

In this study, we investigated the application of NGS in a routine diagnostic workflow in patients with different types of PCD. The goal was to identify a putative correlation between the degree of bone marrow infiltration and the NGS results. We correlated the NGS data with the degree of bone marrow involvement, as identified by cytomorphology (BMC), histopathology (BMH), and multiparameter flow cytometry (MFC) in patients with different types of PCD including MGUS, MM, plasmocytoma and AL-amyloidosis.

## Materials and methods

### Patients

We studied bone marrow (BM) samples from 90 consecutive patients with known or suspected PCD, who underwent a routine BM examination at the University Hospital of Bern between 11/2018 and 05/2020.

Clinical and laboratory details of the patients are listed in Table 1. All patients had signed an informed consent. PCD were classified according to International Myeloma Working Group (IMWG) criteria and current European Society for Medical Oncology guidelines (ESMO, 2017) [11, 14]. Staging and risk assessment were performed according to Myeloma International Staging System (ISS) or Revised Myeloma International Staging System (R-ISS) systems, depending on whether initial cytogenetic data were available [2, 3].

**Table 1:** Baseline clinic-biological characteristics of the patients included

Diagnosis	MGUS	MM	AL-amylosis	Plasmocytoma	Data available
All cases analyzed, %	7 (7.8%)	77 (85%)	4 (4.5%)	2 (2.2%)	90 (100%)
First diagnosis Progression/relapse	7 (100%)	41 (59%) 36 (41%)	3 1	2 0	90 (100%)
Age (years) mean Range	65 (44–82)	63 (32–80)	67 (59 -73)	55	90 (100%)
Sex: Female Male	2 (29%) 5 (71%)	27 (36%) 49 (64%)	1 (25%) 3 (75%)	0 2 (100%)	90 (100%)
FC: % of PC Mean Range	0.70% (0.04–2.6%)	10% (0.002–83%)	0.80% (0.1–1.9%)	1.30% (0.003–2.6%)	90 (100%)
Mutation positive by NGS	2/7 (29%)	60/77 (78%)	1/4 (25%)	1/2 (50%)	90(100%)
Aspirate morphology: % PC Mean Range	5% (3–10%)	44% (3–100%)	14% (10–20%)	10%	90 (100%)
BM biopsy: % PC Mean Range	7% (1–10%)	48% (< 1–100%)	9% (5–15%)	10%	90 (100%)
Β2 microglobuline, mg/L	2.1	5.5	n/a	1.67	57/90 (63%)
M protein, g/dL Mean Range	9.22 (5.38–12)	35.6 (3.10- 367)	n/a	n/a	55/90 (60%)
Free light chains/serum mg/L	32.7 (14–58)	1140 (11–25,000)	56	465	80/90 (88%)
Mutation number by NGS 0 1 > 1	5 (71%) 2 (29%) 0	13 (25%) 25 (47%) 15 (28%)	3 (75%) 1 (25%) 0	1(100%) 0 –	90 (100%)

### Definition of degree of BM involvement by different methods

BM aspirates were routinely analyzed by BMC and MFC, and BM trephine biopsies underwent histopathological and immunohistochemical analyses. Fresh BM aspirates were directly collected for NGS analysis. Additionally, BM samples underwent routine assessment by conventional cytogenetics and fluorescence in situ hybridization (FISH) [12]. Cytogenetic data were available in 63/90 (70%) and high-risk aberrations were detected in nine cases (10%).

To rate the degree of plasma cell (PC) infiltration in the BM aspirates, we defined the following categories for this study: I, < 10%, n = 23 (26%); II, 10–30%, n = 24 (27%); III, > 30%, n = 43 (47%) of PC infiltration.

The PC infiltration in BM biopsies was categorized as: I, < 10% PC (n = 15, 17%); II, 10–30% PC, (n = 29, 32%); III, > 30% PC, (n = 46, 51%).

MFC was performed using Canto-2 flow cytometers (Beckton Dickinson, New Jersey, USA) using a standardized antibody panel for plasma cells (PC) including CD38, CD45 and CD138; cytoplasmic kappa/lambda expression and CD19, CD56, CD20, CD117, CD28, CD27 and CD269 for abnormal/clonal PC classification [23–25]. The degree of PC infiltration, based on the above CD138 + /CD38 + gating strategy was defined as: I < 1% PC, n = 33 (37%); II: 1–3%, n = 20 (23%), III > 3%, n = 37 (40%).

More information is detailed in the Additional file 1.

### *Sample preparation by CD138* + *enrichment*

DNA was extracted after enrichment of PC from fresh BM samples. The enrichment was based on CD138 + magnetic PC sorting (Miltenyi Biotec, Bergisch Gladbach, Germany). Successful enrichment of plasma cells by CD138 + separation was validated using nine samples by MFC and BMC (Additional file 1: Table S1). The median percentage of CD138 + PC before enrichment was 2.6% by MFC and 25% by IHC and 92% (range 72.3%-98.6%) after the enrichment procedure.

### NGS and gene panel design

DNA extraction was performed using the QIAamp DNA mini kit with a QIAcube (Qiagen, Hombrechtikon, Switzerland). The specific panel was designed using the AmpliSeq Designer software (Thermo Fisher Scientific, Reinach, Switzerland). Libraries were prepared with the AmpliSeq™ Library Kit Plus (ThermoFisher Scientific), and the Ion S5 system (ThermoFisher Scientific) was used for sequencing. Bioinformatic analysis was carried out using the Torrent Suite Software 5.6 and IonReporter™ Software 5.6 (ThermoFisher Scientific).The human genome assembly GRCh37 (hg19) from Genome Reference Consortium was used for variant calling. The sensitivity of NGS was limited by a cutoff at 5% variant allele frequency (VAF). The variants were evaluated according to the AMP guidelines. [26]

The NGS panel comprised 15 genes including splice sites or hotspots: *BRAF* (exons 11 and 15), *CCND1*, *DIS3*, *EGR1*, *TENT5C* (*FAM46C*), *FGFR3*, *IDH1* (exon 4), *IDH2* (exon 4), *IRF4* (exon 3), *KRAS* (exons 2 and 3), *MYD88* (L265P mutation), *NRAS* (exons 2 and 3) *PRDM1*, *TP53* and *TRAF3*. The genes and hotspots for the panel were selected according to the frequency of occurrence given in the literature, their prognostic impact, and for some markers their possible function as therapeutic targets (Table 2) [19–21].

**Table 2:** Next generation sequencing panel and mutation frequency

Gene	Criteria for selection for gene panel	Hot spot	Frequency of mutation in PCD	Frequency of mutation in present study	%	Total number of cases	PCD type
*KRAS*	Potentially druggable	Exon 2;3	Up to 30%	24	24%	22	MM
*NRAS*	Potentially druggable	Exon 2;3	Up to 30%	14	16%	14	MM
*DIS3*	Prognosis	Whole gene	Up to 1%	14	12%	11	MM MGUS
*TENT5C* (*FAM46C*)	Prognosis	Whole gene	Up to 4%	13	12%	11	MM MGUS
*TP53*	Prognosis	Whole gene	Up to 20%	9	10%	9	MM
*BRAF*	Potentially druggable	Exon 11; 15	Up to 9% in rr MM*	9	10%	9	MM
*TRAF3*	Frequently mutated	Whole gene	Up to 4%	8	7%	6	MM
*FGFR3*	Prognosis/potentially druggable	Whole	Up to 1.5%	4	6%	4	MM
*IDH1*	Potentially druggable	Exon 4		3	3%	3	MM ALA
*EGR1*	Frequently mutated	Whole gene	Up to 1%	2	2%	2	MM
*IRF4*	Prognosis	Exon 3	Up to 3%	2	2%	2	MM
*CCND1*	Prognosis/potentially druggable	Whole gene	Up to 3%	0	no	0	no
*PRDM1*	Frequently mutated	Whole gene	Up to	0	no	0	no
*IDH2*	Potentially druggable	Exon 4		0	no	0	no

For a random subset of 40 samples, average read depth, uniformity and coverage at 50 reads were collected. The mean values were the following: average read depth18066 reads, uniformity92.7%, coverage at 50 reads: 97.4.

### Statistical analysis

We used R software, version 4.0.2 for statistical analysis. To test for association between PC percentage and number of detected mutations Pearson’s and Spearman’s rank correlations for continuous variables were used. The trend towards a higher PC infiltration in the BM samples when grouped by the number of mutations per sample was analyzed by the Jonckheere-Terpstra test. The Wilcoxon rank sum test was applied to pairwise comparisons of continuous percentages of PC by grouped variables.

Finally, the influence of the number of mutations/sample on overall outcome, as detected by NGS, was tested in a multivariate analysis. A detailed description of the methods is presented in the Additional file 1.

## Results

### Degree of BM involvement by different methods

The study included 77 MM, two solitary plasmacytoma seven MGUS and four AL amyloidosis cases. The mean proportion of infiltrating BM plasma cells was lowest in the seven patients with MGUS: 0.7% (0.04–2.6%) by MFC and 5–7% by BMC (3–10%) and BMH (1–10%); intermediate in four patients with AL-amyloidosis: 0.8% (0.1–1.9%) by MFC and ~ 10% by CM (10–20%) and HM (5–15%); and highest in 77 patients with MM: 10% by MFC (0.002–83%) and 45–50% by BMC (3–100%) and BMH (< 1–100%) (Table 1).

Plasma cell assessment revealed discrepant results in the aspirates and trephine biopsies in nine cases (10%), with PC percentage being higher according to BMH as compared to MFC results, which may be due to peripheral blood dilution of the marrow samples used for MFC or sampling errors [27, 28].

### NGS results: mutation frequency and type

In total, 102 mutations were detected by NGS in 64/90 (71%) cases analyzed. We detected one mutation in 41/90 cases (46%), and more than one mutation per sample in 23/90 (26%) cases with a maximum number of five mutations/sample. In 26 cases (29%), no mutation was detected using our NGS panel.

The proportion of cases affected by mutations was highest in patients with MM (60/77 patients, 78%), and lowest in MGUS with 2/7 (29%) patients showing evidence of mutations. One out of two solitary plasmacytoma cases was positive for *BRAF*V600E (BRAF:c.1799 T > A, p.(Val600Glu)) mutation and one out of four AL amyloidosis cases showed an isolated *IDH1* mutation (Table 2).

The most frequent mutations across all PCD types included members of the *MAPK* signaling pathway – such as *NRAS* and *KRAS* mutations [6, 7, 20]. Mutations in these two genes were only detected in samples from patients with MM and were mutually exclusive, which is in line with previous reports [6, 29]. *KRAS* was mutated in 22/90 samples (24%), occurring as an isolated mutation in 12 cases (55%). In two cases, two different mutations of *KRAS* were identified. Gly12, Gln61, and Gly13 were the most frequently mutated residues, representing the known major and two minor hotspots in *KRAS* [6, 20, 30]. *NRAS* and *DIS3* were identified as the second most recurrently mutated genes, each of them detected in 14/90 (16%) and 11/90 (12%) of cases, respectively. In 8/14 cases (57%), *NRAS* was the only mutation detected. In contrast, mutations in *DIS3* were almost always (10/11 or 91%) accompanied by other mutations, representing a unique mutation/sample in one case only (MGUS).

Mutations of *FAM46C* (*TENT5C*) were the third most frequent mutations in the cohort: 14 mutations were observed in 11 patients (12%)*. FAM46C* (*TENT5C*) mutations were found as isolated molecular alterations in six out of 11 cases (55%).

*TP53* and *BRAF* mutations were identified in nine samples (10%) each. *TP53* was an isolated mutational event in 2 out of 9 cases only, and *BRAF* in 7 out of 9 (78%) of cases. *TP53* mutations were seen exclusively in MM cases and showed a high frequency (6 out of 7 cases) in patients investigated due to relapse or progression of the disease, in accordance with previously published results [16, 31–33]. Regarding *BRAF*, in most cases (6/9, 67%) the activating “classic” *V600E* type I mutation (BRAF:c.1799 T > A, p.(Val600Glu)) was detected, in one case the type II G469A mutation (BRAF:c.1781A > G, p.(Asp594Gly)) or kinase-dead (*D594A,* BRAF:c.1406G > C, p.(Gly469GAla)) type of alteration was identified, respectively [8, 30, 34].

*TRAF3* was affected in six MM cases (7%) and was found to be an isolated mutation in 2 out of 6 cases. Four cases showed *FGFR3* mutations (4.5%), all with a high-risk t(4;14) translocation. This association has already previously been reported [35]. *IDH1* was mutated in three cases (3%) at the hot spot position R132. *IRF4* and *EGR1* mutations were detected in two (2%) samples each. In both cases this correlated with at least one other mutated gene. For the remaining genes on the panel, namely *CCND1*, *IDH2*, *MYD88* and *PRDM1*, no mutations were observed in this cohort.

### Correlation between NGS results and degree of bone marrow infiltration by clonal PC

**Fig. 1:**
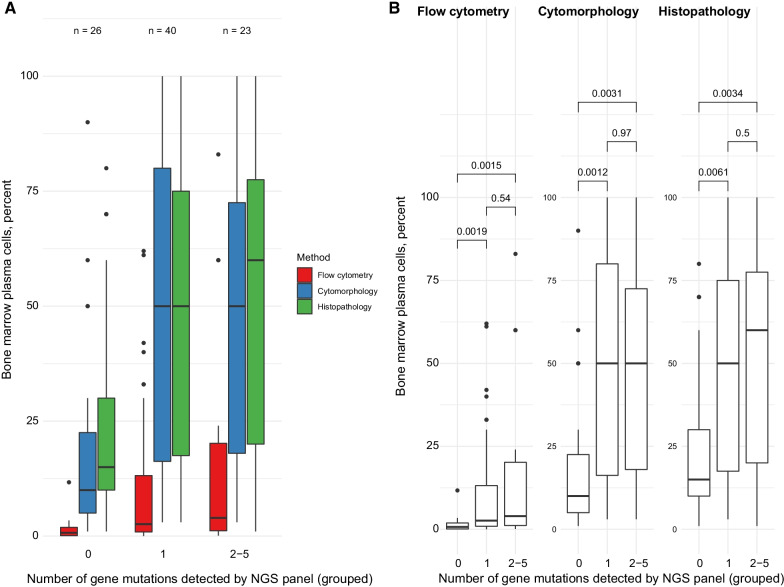
**a** Correlation between degree of BM infiltration by all methods and mutation number by NGS. **b** Comparison of bone marrow plasma cell infiltration by groups of the number of gene mutations detected by NGS panel. Data are shown for each method of bone plasma cell count. Numbers denote *p*-values of pairwise Wilcoxon test

Our analyses suggested that in these three groups, the degree of BM infiltration was the highest in samples with more than one mutation/sample with a mean of 15% aberrant PC as defined by MFC (0.1–83%), and ~ 50% (range 1–100%) PC as defined by both BM cytology and histology (Fig. 1). In samples with one mutation, identified by NGS, the pathologic PC percentage by flow cytometry was lower (mean 11%; range 0.002–62%), but similar (~ 50%, range 3–100%) as defined by both BMC and BMH.

We observed the lowest PC infiltration in BM samples with no mutation detected. The mean PC infiltration in this group was only 1.6% by MFC (0.003–11%), 20% by BMC (1–90%), and 24% by BMH (1–80%) (Fig. 1).

Those findings were further confirmed by positive Jonckheere-Terpstra test (*p* = 0.003). The pairwise comparison also showed a significant difference between samples without mutations and both (1) samples with one mutation per sample (*p* = 0.006), and (2) samples with more than one mutation per sample (*p* = 0.003) (Fig. 1).

The number of mutations directly influenced outcome, as was confirmed by multivariate analysis with the odds ratio (OR) for PC infiltration being 1.023 (95% CI 1.008, 1.038; *p* = 0.002). For comparison of various prediction’s models, see Additional file 1: Fig S1–3.

### Correlation of NGS data and clinical/biological parameters

We performed correlation analyses between the presence and number of mutations per sample with the clinical and biological parameters of the 90 patients in the cohort, as presented in Table 3.

**Table 3:** Summary statistics of bone marrow plasma cell infiltration grouped by method and number of mutations

Mutation load/type	PCD type	Disease relapse/progression	Serum light chains mean mg/L, range	M-gradient mean g/L, range	β2, mean mg/L, range
No mutation N = 26	MGUS = 5 ALA = 3 MM = 17	7/26 (27%)	611 (14.3–2830)	13 (3–60.0)	4.2 (0.32–17.42)
1 mutation N = 41	MGUS = 2 ALA = 1 IP = 1 MM = 37	15/41–37%	737 (11.0–8320)	30 (4.8–72.4)	4.6 (1.55–16.7)
> 1 mutation N = 23	MM = 23	15/23–65%	1773 (6.2–25,000)	49 (3.70–367.0)	6.7(1.8–37)

Most samples without mutation (26 cases, 29%) were from patients undergoing initial staging (19/26 cases or 73% of this subgroup), whereas BM samples with > 1 mutation (23 cases, 26%) were mostly acquired from patients with relapsed/progressive disease (15/23 cases, or 65% of this subgroup).

The level of biological markers (mean), reflecting the tumor burden, progressively increased from MGUS to MM cases as well as from cases with no mutation to those with ≥ 1 mutation/sample.

## Discussion

First, we could confirm the feasibility of NGS in patients with PCD as a part of the routine diagnostic procedure. We observed no major technical obstacles with sample collection and quality of DNA preservation in BM samples stored up to 30 h (for instance, over the weekend) at room temperature. In particular, CD138 + based PC enrichment of the samples was a quick and reliable procedure to obtain the maximum percentage of PC for subsequent DNA extraction.

Second, the pattern of genomic lesions, observed across the PCD cases analyzed, was largely in line with previous NGS studies with the majority of alterations detected in *KRAS*, *NRAS*, *DIS3*, *TENT5C* (*FAM46C*), *TP53* and *BRAF* genes [7, 8, 18, 21, 30, 36].

Remarkably, we observed a significant correlation between the degree of BM involvement, as detected by phenotypic methods (BMC, BMH and MFC), and the likelihood of recognizing a mutation by NGS (Fig. 1). Hypothetically, this may be due to a higher chance of molecular evolution with acquisition of novel mutations in cases with a higher myeloma cell load. In our study, the probability identifying a mutation by NGS in BM material was highest in samples with more than 10% clonal PC, as assessed by flow cytometry, or with at least 20% of PC, as detected by BMC or BMH. However, this correlation was only detected at initial diagnosis of PCD/MM. In PCD and especially MM, disease progression is frequently associated with molecular clonal evolution and higher rates of mutations, as compared to the primary manifestation [15]. Since the probability of detecting mutations is higher with suspected MM relapse or progression, all BM samples from such patients should, theoretically, be evaluated by NGS, regardless of the degree of infiltration. So far, no specific genomic lesions appear clinically or prognostically important in MGUS. However, more studies to determine the importance of detection of genomic alterations in MM progression are needed, before definite conclusions can be made, and further studies are needed to confirm the above considerations. Similarly, the clinical and/or prognostic value of a positive or negative mutational status by NGS for patients with plasma cell dyscrasias needs to be further clarified considering the limitations of the present study.

It should be emphasized that we used CD138 + based PC sorting. Indeed, the pre-analytic DNA quantity was lower in samples with inferior plasma cell infiltration, but still sufficient for the NGS analysis. As described in the results section, the enrichment procedure permitted equalizing the percentage of CD138 + PC in all analyzed samples as high as 90% (median).

With the balanced CD138 + PC content obtained from all samples, a DNA quantity bias (more PC more DNA higher probability of detection of mutations by NGS) seems less likely. Therefore, if NGS results directly correlate with the degree of BM infiltration, the question arises, whether the presence of molecular mutations by NGS in BM samples reflects the mutational burden of the whole tumor mass in PCD [37]. The clonal PC development undergoes an important quantitative and qualitative diversity at different time points [6, 7, 18]. Accordingly, a low BM tumor burden at the initial stages of PCD formation would probably not allow for identification of very small subclones that already harbor genomic aberrations. The question of adequacy of BM analysis for the assessment of the whole tumor genomic in PCD has already been raised [38]. The possible limitation of BM sampling site and plasma cell quantity bias should be further evaluated in studies on comparative genomic analysis of the liquid malignant DNA component in peripheral blood (liquid biopsies) and that of BM samples from patients with different PCD.

Concerning genomic data from this study, as expected, the mutational complexity was higher in MM than in MGUS or AL-amyloidosis. MM cases were positive in two thirds or 77% with a maximum of up to five mutations per sample detected (Table 1). This observation supports the idea of progressive changes of the mutational complexity during PCD progression from MGUS to MM with a hierarchical time-dependent structure of the genomic lesions [7, 36, 39, 40]. Both mutations seen in our two MGUS cases affected the *DIS3* and *TENT5C* (*FAM46C*) genes. According to the available literature, mutations in these two potential tumor suppressor genes, especially *DIS3*, occurred later during the transition from MGUS to MM [36, 41].

Interestingly, in most of the cases with one mutation per sample, *NRAS*, *KRAS*, or *BRAF* mutations were detected. Since mutations in these genes have mostly been described to occur as secondary driver events, their presence may indicate PCD/MM progression by intramedullary tumor expansion and invasion. Indeed, activation of the MAPK signaling pathway was found to be associated with advanced BM spreading of MM cells mediated by osteoblast activation and stimulation of matrix metalloproteinase expression [42]. In NGS based studies, Rossi et al. suggested a probable role of activating mutations in *NRAS*, *KRAS* or *BRAF* genes for PCD progression from MGUS to smoldering and symptomatic myeloma [36]. Finally, the association between mutated *TP53* and relapsed or refractory MM in our study confirms the important role of continuous intraclonal mutational MM evolution for disease progression and advanced intramedullary spreading [19, 31, 36].

In addition, M-gradient level was the only parameter to differ significantly between cases with and without mutation as detected by NGS (Table 1).

## Conclusions

Our study confirms the feasibility of NGS in patients with PCD as a part of the routine diagnostic procedure. By an NGS panel designed for plasma cells dyscrasias comprising 15 genes and hotspots, which we performed after CD138 + plasma cell enrichment, we were able to confirm a high rate of mutations especially in MM cases. We observed a significant positive correlation between the degree of BM involvement, as detected by phenotypic methods (BMC, BMH and MFC), and the likelihood of recognizing a mutation by NGS. Furthermore, the probability of a positive mutation status was higher at relapse/progression than at initial diagnosis. It is very likely, that by enlarging the NGS panel and including less frequently observed mutational variants, the proportion of NGS “negative” cases will be further reduced. The clinical and prognostic impact of targeted NGS panels designed for patients with PCD should be further studied, favorably within larger prospective cohorts.

## Supplementary Information

**Additional file 1**. Supplementary tables and figures.

## Data Availability

The data presented in this study are available on request from the corresponding author. The data are not publicly available due to institution-related patient identity restrictions.

## Abbreviations

PCD: Plasma cell dyscrasias
PC: Plasma cells
MM: Multiple myeloma
MGUS: Monoclonal gammopathy of unclear significance
NGS: Next-generation sequencing
BM: Bone marrow
BMC: Cytomorphology
BMH: Histopathology
MFC: Multiparameter flow cytometry
IMWG: International myeloma working group
ESMO: European society for medical oncology
ISS: Myeloma international staging system
R-ISS: Revised myeloma international staging system
FISH: Fluorescence in situ hybridization
