# Supplementary material for: Correlation of plasma cell assessment by phenotypic methods and molecular profiles by NGS in patients with plasma cell dyscrasias
Source: BMC Med Genomics. 2022 Sep 23;15:203. doi: 10.1186/s12920-022-01346-1 (PMC9503268; doi:10.1186/s12920-022-01346-1)

**Additional File**

**Correlation of plasma cell assessment by phenotypic methods and molecular profiles by NGS in patients with plasma cell dyscrasias**

Ekaterina Rebmann Chigrinova^1^*, Naomi A. Porret^1^*, Martin Andres^1^, Gertrud Wiedemann^1^, Yara Banz^2^, Myriam Legros^3^, Matthias Pollak^1^, Elisabeth Oppliger Leibundgut^1^, Thomas Pabst^4^**, Ulrike Bacher^1,3^**

* equal contribution; should both be considered as first authors;

** equal contribution; should both be considered as last authors.

^1^ Department of Hematology, Inselspital, Bern University Hospital, University of Bern, Bern, Switzerland

^2^ Institute of Pathology, University of Bern, Bern, Switzerland

^3^ Center for Laboratory Medicine (ZLM), Inselspital, Bern University Hospital, University of Bern, Bern, Switzerland

^4^ Department of Medical Oncology, Inselspital, Bern University Hospital, University of Bern, Bern, Switzerland

**Methods**

***Clinical patient’s assessment***

The initial evaluation of CRAB criteria was performed based on a complete blood cell count, serum creatinine, creatinine clearance, and routine clinical chemistry. The routine diagnostic procedures included evaluation of the monoclonal (M) component by serum and/or urine protein electrophoresis (concentrate of 24h urine collection); nephelometric quantification of serum IgG, IgA and IgM immunoglobulin level; characterization of the heavy and light chains by immunofixation; and serum-free light-chain (FLC) measurement. All patients had IRM or whole-body low-dose computed tomography (WBLD-CT) for diagnosis of lytic disease. 18F-fluorodeoxygluscose positron emission tomography with CT (PET-CT) to evaluate bone lesions was performed if indicated.

***Bone marrow samples processing***

Bone marrow cytology (BMC) and visual assessment of the plasma cells (PC) were performed on aspirate smears stained according to standard May-Gruenwald-Giemsa (MGG) method. For BM histology (BMH), BM biopsies were processed following fixation in 10% neutral buffered formalin and overnight decalcification and processing in the Logos Tissue Processor (Biosystems, Zullwil, Switzerland). Standard laboratory stains included hematoxylin and eosin stain, Giemsa stain and silver staining for fibres. Immunohistochemical stains were performed on the fully automated stainer BOND-III systems (Leica Biosystems) using the following markers: CD138, CD20, CD56 and Cyclin D1. PC clonality was assessed by kappa/lambda light chain expression profiles and analyses of the heavy chains IgG, IgM and IgA was routinely performed.

***CD138+ enrichment and NGS panel design***

CD138+ magnetic cell sorting has been performed with the commercially available system consisting of the Whole Blood Column Kit, the Whole Blood and Bone Marrow CD138 MicroBeads or MACSprep Multiple Myeloma CD138 MicroBeads combined with the autoMACS® Pro Separator was used with an initial filtration step via 100 µm MACS SmartStrainers (Miltenyi Biotec, Bergisch Gladbach, Germany).

**Table S1. Plasma cell counting in nine BM samples before and after enrichment**

|  | % CD138+ PC before enrichment | | | % CD138+ PC  after  separation (by MFC) |
| --- | --- | --- | --- | --- |
|  | **by MFC** | **by BMC** | **by BMH** |  |
| Average | 3.8 | 22.0 | 39 | 87.4 |
| Min | 0.3 | 0.1 | 8 | 72.8 |
| Max | 11.6 | 50 | 80 | 96.2 |
| Median | 2.6 | 7 | 25 | 92.1 |

**Statistical analysis, methods description**

We used R software, version 4.0.2 for statistical analysis. In order to see, if there is any statistically powerful association between the probability of mutation detection by NGS and the degree of PC infiltration. PC percent was taken as a continuous variable. The PC percentage was analyzed separately for each of the three methods used (BMC, BMH or MFC). The number of mutations, detected by NGS was also considered as a continuous variable or grouped into three categories, according to the number of mutation per sample: no mutations (“0”), one mutation (“1”) and more than one mutation (“>1”). To test for association between PC percentage and number of detected mutations we used Pearson’s and Spearman’s rank correlations for continuous variables. The trend toward a higher PC infiltration in the samples when grouped by the number of mutation per sample (“0”, “1”, “>1”) was analyzed by the Jonckheere-Terpstra test. The Wilcoxon rank sum test was used to pairwise comparisons of continuous percentages of PC by grouped variables.

Additionally, we stratified the samples by available clinical patient’s parameters as sex, age and disease status (initial staging versus relapse/refractory disease). For multivariate analysis, we defined the number of detected mutations as the outcome variable, while PC percent was defined as predictor of interest; sex, age group and disease state were taken as covariates. Generalized linear regression models were set up for number of detected mutations as ordinal variable (proportional odds logistic regression model) and as nominal variable (multinomial logistic regression model). PC percent and the three stratification variables as well as interaction terms were included in the full model. Variables were omitted by stepwise backward selection to find suitable models. Pointwise 95 % Wald type confidence intervals were calculated for the effect of PC percentage.

**Figure S1. A proportional odds logistic regression model**

Grouped number of gene mutations (0, 1, and 2-5) was the ordinal outcome. The full model contained bone marrow plasma cell (BMPC) fraction estimated by histopathology, disease state (at diagnosis, versus during follow-up), age group and sex including first order interaction terms. In the final model, obtained from stepwise backward selection, BMPC, disease state, age group and the interaction term between disease state and age group was retained while sex and the other interaction terms were dropped. BMPC effect estimate: OR = 1.022 (95% CI 1.008, 1.038). A: effects of BMPC, stratified by age group and disease state, solid lines: estimate of effect, dotted line: pointwise 95 % confidence interval estimate. B: stacked area plot.


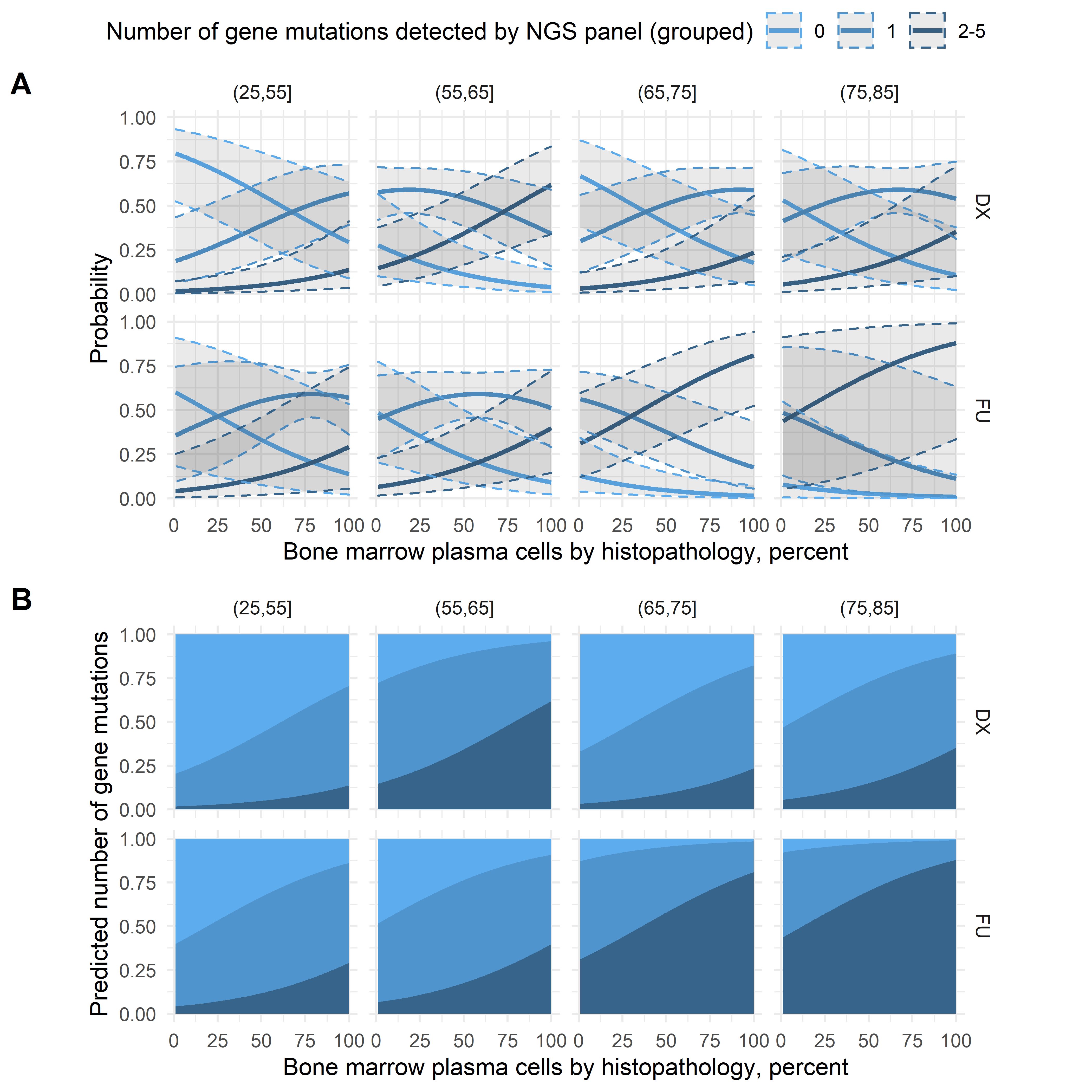


**Figure S2. Multinominal model**

To assess effects of BMPC on probability of the number of gene mutations without proportional odds assumption, a multinominal model was fitted. Grouped number of gene mutations (0, 1, and 2-5) were taken as nominal outcome. Similar to the proportional odds logistic regression model, the full model contained bone marrow plasma cell (BMPC) fraction estimated by histopathology, disease state (DX, FU), age group and sex including first order interaction terms. In the final model, obtained from stepwise backward selection, again, BMPC, disease state, age group and the interaction term between disease state and age group were retained while sex and the other interaction terms were dropped. With 0 mutations as reference group, OR for 1 mutation is 1.031 (95% CI 1.010, 1.053) and for 2-5 mutations 1.038 (95% CI 1.012, 1.065), respectively. A: effects of BMPC, stratified by age group and disease state, solid lines: estimate of effect, dotted line: pointwise 95 % confidence interval estimate. B: stacked area plot.


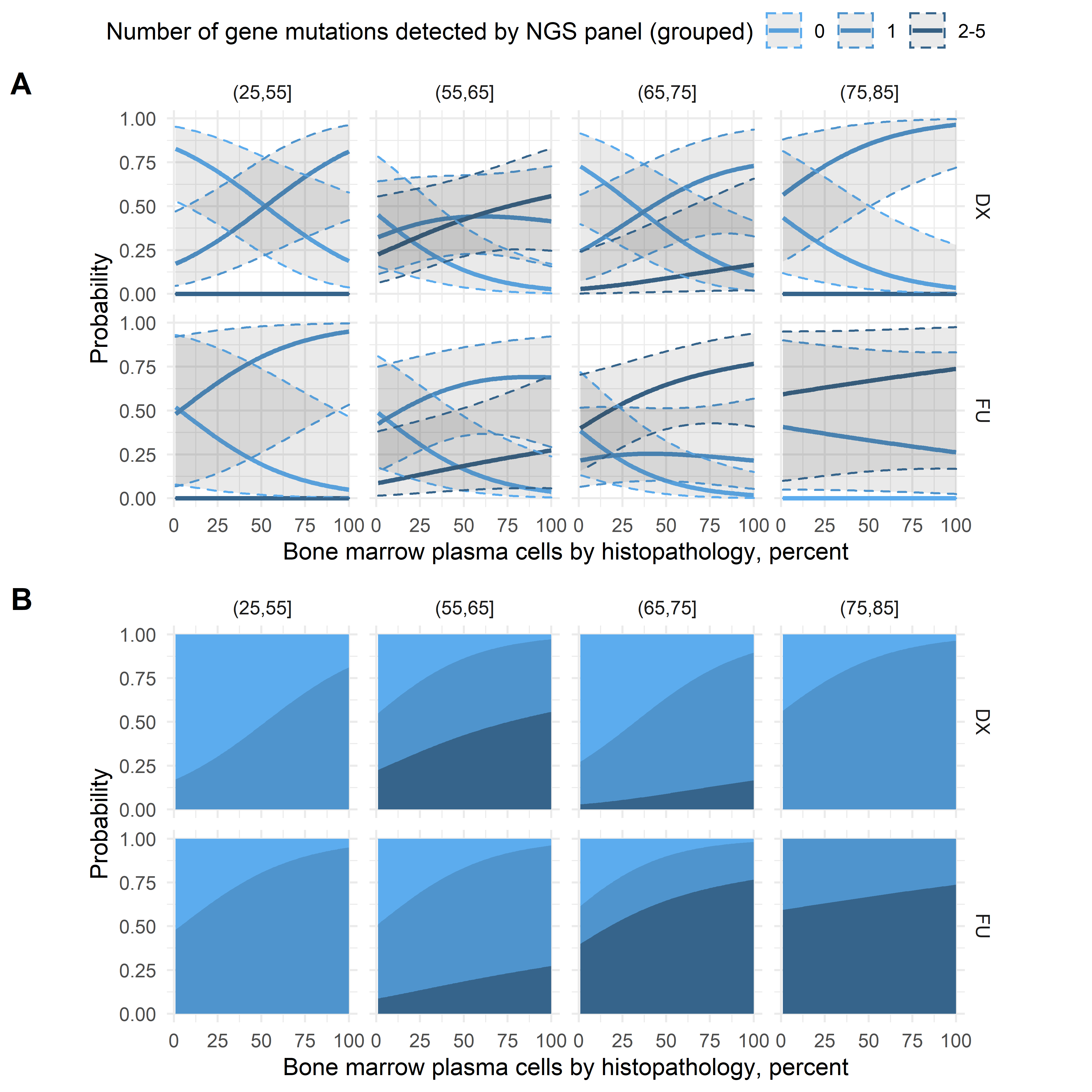


**Figure S3. Receiver operator curves (ROC)**

Crude: BMPC as direct predictor. DX: BMPC as direct predictor for the subset of cases with disease stage “DX”. FU: BMPC as direct predictor for the subset of cases with disease state “FU”. full2: logistic regression model including BMPC, disease state, age group, sex and first order interaction terms as predictors. set: logistic regression model including the predictors BMPC and set. set_age: logistic regression model with predictors BMPC and disease state.


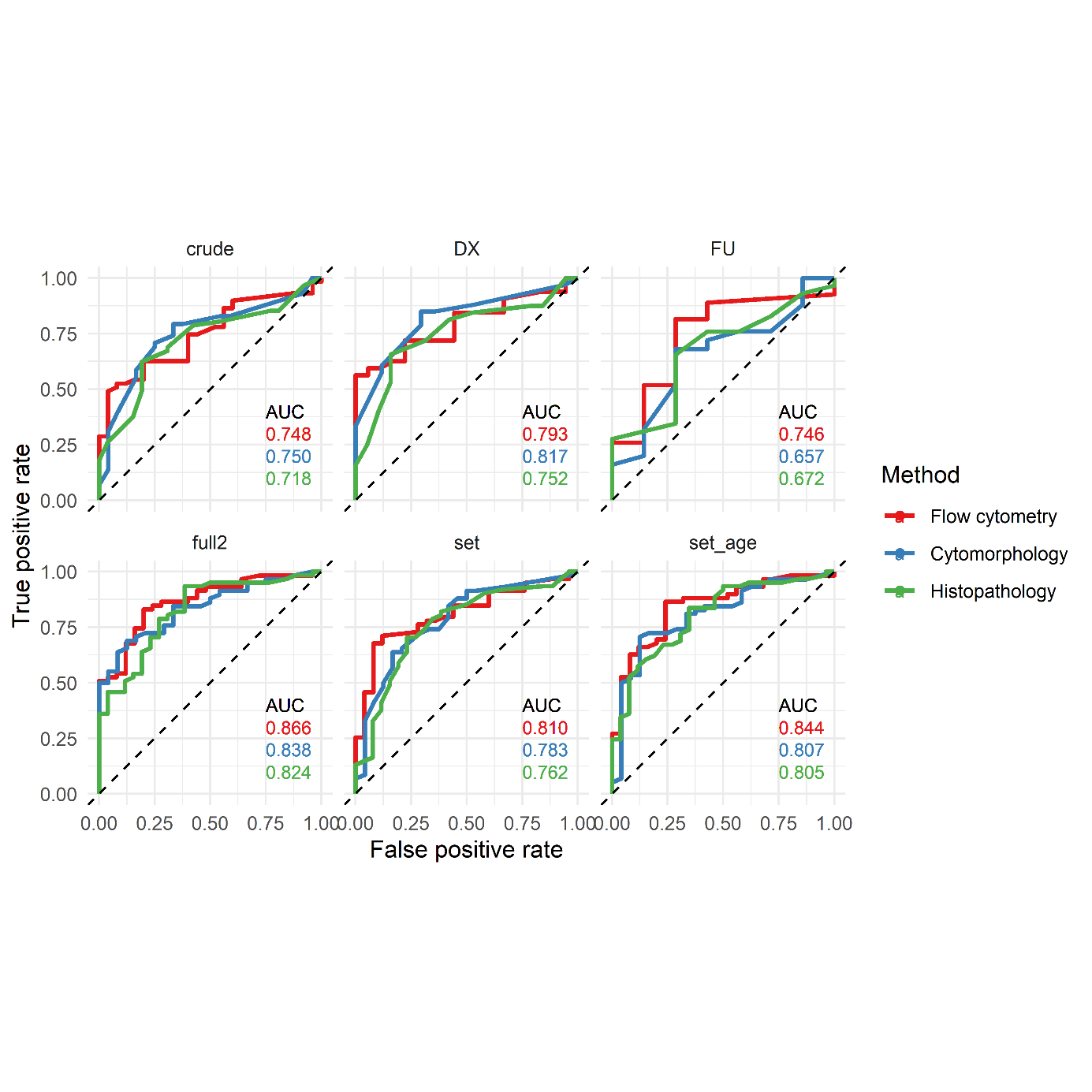

Supplement: Supplementary file 1 — Additional file 1. Supplementary tables and figures. [file 12920_2022_1346_MOESM1_ESM.docx]
